# Magnetite nanoparticles for functionalized textile dressing to prevent fungal biofilms development

**DOI:** 10.1186/1556-276X-7-501

**Published:** 2012-09-06

**Authors:** Ion Anghel, Alexandru Mihai Grumezescu, Ecaterina Andronescu, Alina Georgiana Anghel, Anton Ficai, Crina Saviuc, Valentina Grumezescu, Bogdan Stefan Vasile, Mariana Carmen Chifiriuc

**Affiliations:** 1 Carol Davila University of Medicine and Pharmacy, Bucharest, 50474, Romania; 2 Faculty of Applied Chemistry and Materials Science, Politehnica University of Bucharest, Bucharest, 011061, Romania; 3 ENT Clinic, Coltea Hospital, Carol Davila University of Medicine and Pharmacy, Bucharest, 030171, Romania; 4 Faculty of Biology, University of Bucharest, Bucharest, 060101, Romania

## Abstract

The purpose of this work was to investigate the potential of functionalized magnetite nanoparticles to improve the antibiofilm properties of textile dressing, tested *in vitro* against monospecific *Candida albicans* biofilms. Functionalized magnetite (Fe_3_O_4_/C_18_), with an average size not exceeding 20 nm, has been synthesized by precipitation of ferric and ferrous salts in aqueous solution of oleic acid (C_18_) and NaOH. Transmission electron microscopy, X-ray diffraction analysis, and differential thermal analysis coupled with thermo gravimetric analysis were used as characterization methods for the synthesized Fe_3_O_4_/C_18_. Scanning electron microscopy was used to study the architecture of the fungal biofilm developed on the functionalized textile dressing samples and culture-based methods for the quantitative assay of the biofilm-embedded yeast cells. The optimized textile dressing samples proved to be more resistant to *C. albicans* colonization, as compared to the uncoated ones; these functionalized surfaces-based approaches are very useful in the prevention of wound microbial contamination and subsequent biofilm development on viable tissues or implanted devices.

## Background

Most of the organisms responsible for device-related infections including central venous catheters, joint devices, dialysis access devices, cardiovascular devices, urinary catheters, penile implants, voice prostheses, dentures, and ocular implants can grow in polysaccharide-rich extensive biofilms and are associated with drastically enhanced ability to express resistance against most antimicrobial agents [1-3]. *Candida* species are the most common fungi isolated from device-related infections, inquiring sometimes the removal of the device [4-6]. However, when *Candida albicans* enters sterile cavities or tissues and causes infection, treatment may be difficult and prolonged. Our previous studies have demonstrated that the *Rosmarinus officinalis* essential oil-coated magnetic nanoparticles strongly inhibited the adherence ability and biofilm development of *C. albicans* and *Candida tropicalis* clinical strains [7] on the catheter surface, and usnic acid-coated magnetic nanoparticles strongly inhibited the adherence ability and biofilm development of *Staphylococcus aureus* on the coverslips surface, opening new perspectives for the design of antimicrobial and antibiofilm surfaces based on hybrid functionalized nanostructured biomaterials [8].

Nanotechnology deals with the science and technology at dimensions of roughly 1 to 100 nm, although 100 nm presently is the practically attainable dimension for textile products and applications. The technology can be used in engineering-desired textile attributes, such as fabric softness, durability, and breathability and in developing advanced performance characteristics; namely, water repellency, fire retardancy, and antimicrobial resistance in fibers, yarns, and fabrics. The enhancement of textile materials by nanotechnology is expected to become a trillion-dollar industry in the next decade, with tremendous technological, economic, and ecologic benefits. Although textile industry is a small part of the global research in the emerging areas of nanotechnology, the fibers and textiles industries in fact were the first to have successfully implemented these advances and demonstrated the applications of nanotechnology for consumer usage [9].

The nanoparticles have been largely used for different biomedical applications, such as targeted drug delivery [10-12], magnetic resonance imaging [13], alternative drug and vaccine delivery mechanisms (e.g., inhalation or oral in place of injection), bone growth promoters, cancer treatments [14], biocompatible coatings for implants [15], sunscreens (e.g., using ZnO and TiO_2_)/cosmetics [16-18], biolabeling and detection (e.g., using Au) [19], carriers for drugs with low water solubility, fungicides (e.g., using ZnO), magnetic resonance imaging contrast agents (e.g., using superparamagnetic iron oxide), new dental composites, biological binding agents (e.g., for high phosphate levels), antivirals, antibacterials (e.g., Ag) [20], anti-spore nonchemical creams, and powders (using surface tension energy on the nanoscale to destroy biological particles) [21]. We have previously reported that magnetite nanosystems exhibited antimicrobial activity against planktonic, as well as adherent microbial cells [22]. In this study, we assessed the potential of Fe_3_O_4_/C_18_ nanoparticles to improve the *in vitro* antibiofilm properties of textile dressings, using a monospecific fungal biofilm experimental model. The changes induced by the presence of nanoparticles in the *C. albicans* biofilm formation on textile dressing samples were assessed by quantitative, culture-based methods for viable cell counts (VCCs) assay, and qualitative analysis was performed by scanning electron microscopy (SEM).

## Methods

### Preparation of functionalized magnetite nanoparticles

Functionalized magnetite nanoparticles are usually prepared by wet chemical precipitation from aqueous iron salt solutions by means of alkaline media, like NaOH or NH_3_[23,24]. In the present paper, the functionalized magnetite nanoparticles were prepared by a modified precipitation method. Half gram of oleic acid was solubilized in a known volume of distilled-deionized water, corresponding to a 0.01% (*w/w*) solution, under stirring conditions (200 rpm) at 25°C. Then, 2 g of NaOH was added to the oleic acid solution. Thereafter, a 500-mL solution, consisting of 0.6 g of FeCl_3_ and 1 g of FeSO_4_ × 7H_2_O was dropped under permanent stirring up to pH = 8, leading to the formation of a black precipitate. The product was repeatedly washed with methanol and subsequently dried in an oven at 60°C until reaching a constant weight.

### Characterization

#### TEM

The transmission electron microscopy (TEM) images were obtained on finely powdered samples using a Tecnai™ G2 F30 S-TWIN high-resolution transmission electron microscopy from FEI (FEI Company, Hillsboro, OR, USA) equipped with area electron diffraction and selected area electron diffraction. The microscope was operated in transmission mode at 300 kV with TEM point resolution of 2 Å and line resolution of 1 Å. The finely micronutrient powders was dispersed into pure ethanol and ultrasonicated for 15 min. After that the diluted sample was put onto a holey carbon-coated copper grid and left to dry before it was analyzed through TEM.

#### XRD

X-ray diffraction analysis (XRD) was performed using a Shimadzu XRD 6000 diffractometer (Shimadzu Corporation, Chiyoda-ku, Tokyo, Japan) at room temperature. In all the cases, Cu Kα radiation from a Cu X-ray tube (run at 15 mA and 30 kV) was used. The samples were scanned in the Bragg angle 2θ with the range of 10 to 80.

#### DTA-TG

The differential thermal analysis (DTA) coupled with thermo gravimetric analysis (TGA) was performed with a Shimadzu DTG-TA-50 H, at a scan rate of 10°C/min, in air.

### Textile dressing coating

In order to obtain the coated textile dressing, the textile material recommended for otomastoiditis, nasal-sinus, and cervical wound dressing (obtained from the local provider of ENT Coltea Bucharest Hospital) was aseptically cut in samples of 1 cm^2^, submerged in the obtained nanofluid (Fe_3_O_4_/oleic acid/CHCl_3_ 0.33% (*w/v*) under magnetic field, and extemporaneously dried (due to the convenient volatility of chloroform) [7].

### Fungal biofilm model

The artificial monospecific biofilms were developed using *C. albicans* strains recently isolated from the clinical specimens identified by the Vitek 2 automatic system (bioMérieux Inc., Durham, NC, USA) and previously tested for their susceptibility to currently used antifungals (voriconazole, itraconazole, caspofungin, amphotericin B, fluconazole, and flucytosin), as well as to some essential oils [25].

### Microbial adherence to uncoated/coated textile dressing

The microbial adherence ability was investigated in six multiwell plates in which sterile textile dressing samples of 1 cm^2^ with and without nanoparticles coating have been placed. The plastic wells were filled with sterile liquid culture medium, inoculated with 300 μL of 0.5 McFarland microbial suspensions and incubated for 72 h at 30°C. At 24 h after the culture medium was removed, the textile samples were washed three times in phosphate buffered saline (PBS) in order to remove the nonadherent strains, and fresh glucose broth was added. Also, VCCs assay have been achieved for both working variants (uncoated textile dressing and textile dressing with magnetic nanoparticles coating) at 48 h in order to assess the fungal biofilm developed on these substrata. In this purpose the fungal cells that adhered to the textile samples have been removed by vortexing, and brief sonication in a fixed volume of sterile saline was performed, and serial dilutions ranging to 10^−1^ from 10^−4^ have been spotted on Sabouraud agar and incubated for 24 h at 30°C. The colony-forming units have been numbered and multiplied by the dilution factor, and the spotted volume correction was done in order to obtain the VCCs [26].

### Examination of the biofilm architecture by SEM

In order to evaluate the architecture of the fungal biofilm developed on uncoated and magnetite-based textile dressing, SEM was used. After 48 h of incubation, the samples were removed from the plastic wells, washed three times with PBS, fixed with cold methanol, and dried before microscopic examination. SEM analysis was performed on a HITACHI S2600N electron microscope (Hitachi High-Tech, Minato-ku, Tokyo, Japan) at 25 keV in primary electrons fascicle, on samples covered with a thin silver layer.

## Results and discussion

**Figure 1:**
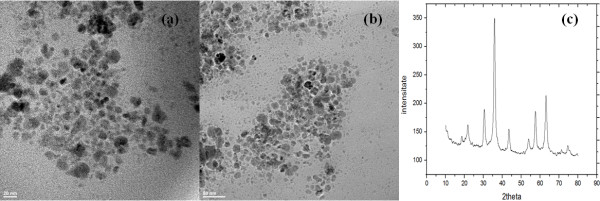
**TEM images and XRD pattern of Fe**_**3**_**O**_**4**_**/C**_**18**_**nanofluid.**

**Figure 2:**
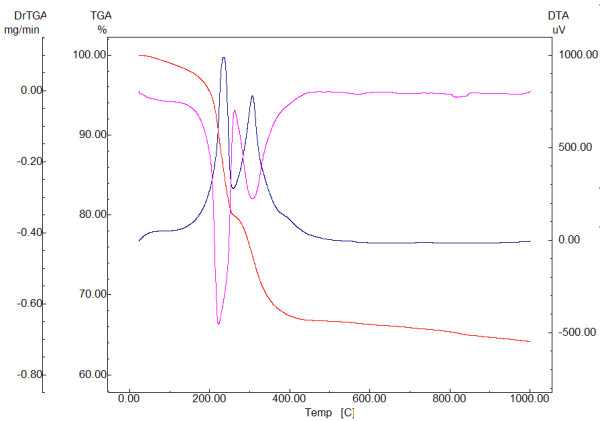
**DTA-TG analysis of Fe**_**3**_**O**_**4**_**/C**_**18**_**.**

In order to interpret the obtained results, the TEM images, XRD pattern, and DTA-TG analysis were recorded and presented in Figures 1 and 2.

Figure 1a,b shows the TEM images of Fe_3_O_4_/C_18_. The observed morphology of nanoparticles is spherical, without aggregation of particles. The TEM micrograph suggests that the particle diameter ranged from 5 to 20 nm.

The X-ray diffraction pattern of the Fe_3_O_4_/C_18_ is shown in Figure 1c. The magnetite peaks were identified in the sample as the only crystalline phase (the main peaks of the magnetite are centered at 2θ = 30.31, 35.71, 43.31, 57.61, and 62.81). Based on the intensity of the characteristic peaks of magnetite, no preferential directions of crystallization were identified [27].

The wet synthesis of these Fe_3_O_4_/C_18_ nanoparticles leads to the retention of approximately 2.84% humidity which is lost at heating (below 180°C). The three exothermic peaks centered at 234°C, 306°C, and 388°C can be attributed to the organic phase decomposition. The total loss of the oleate shell decomposition is determined as mass loss between 180°C and 450°C which is being estimated as 30.25%. The total weight loss of the Fe_3_O_4_/C_18_ sample is 35.79%, while in the case of the pure Fe_3_O_4_ sample, this is only 5.55%. Assuming that all the oleate is decomposed up to 1,000°C, it can be estimated that the weight ratio Fe_3_O_4_:C_18_ is approximately 2:1. Also, in this paper we have investigated the potential magnetic nanoparticles to improve the antibiofilm properties of a textile dressing using an *in vitro* model of monospecific *C. albicans* biofilm.

**Figure 3:**
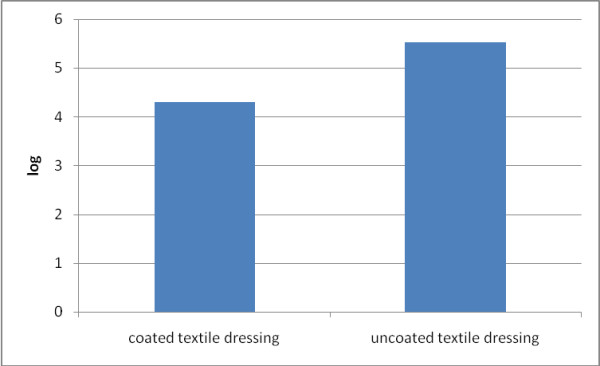
**The logarithmic values of viable cell counts of fungal cells.** The logarithmic values of viable cell counts of fungal cells which adhered and embedded in biofilms and formed on the textile dressing surface (uncoated versus coated textile dressing).

The quantitative assay of viable fungal cells embedded in the biofilm and developed on the textile dressing samples revealed more than 1 log reduction of VCCs in the case of coated textile dressing, which is functionalized with magnetic nanoparticles coating as compared to the uncoated sample (Figure 3), which served as a control in our experiments. The previous studies demonstrated that nanoparticles show promise when applied as a coating to the surface of protective clothing in reducing the risk of transmission of infectious agents. Li et al. demonstrated that nanoparticle-coated facemasks (nanoparticles consisting of a mixture of silver nitrate and titanium dioxide) induced a 100% reduction in the viable *Escherichia coli* and *S. aureus* observed in the coated mask materials after 48 h of incubation. Skin irritation was not observed in any of the volunteers who wore the facemasks [28]. Although different studies have established the microbicidal effect of different metallic nanoparticles against different types of microorganisms, their mechanism of action has not been clearly elucidated. However, a lot of possible mechanisms have been formulated by different researchers; i.e., (1) the nanoparticles attachment to the surface of the cell membrane, disturbing its function and inducing the depletion of adenosine triphosphate levels, a molecule that is the principal form of energy immediately usable by the cell; (2) penetration and intracellular release of metallic ions into the cells, targeting protein, nucleic acid, and cell wall synthesis [29-31]; (3) transition metals (iron, copper, chromium, vanadium, etc.) nanoparticles can generate reactive oxygen species acting as catalysts in Fenton type reactions; for example, the reduction of hydrogen peroxide with ferrous iron results in the formation of hydroxyl radical that is extremely reactive, attacking biological molecules situated within diffusion range [32]; and (4) as shown in several studies, small nanoparticles are also able to enter the mitochondria and produce physical damage, contributing to the oxidative stress [33].

**Figure 4:**
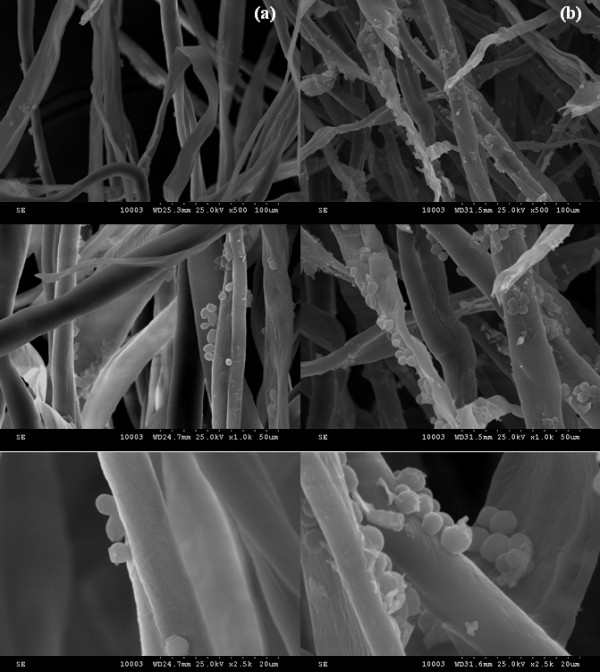
SEM micrographs of coated (a) and uncoated (b) textile dressing.

The SEM examination of the uncoated textile dressing samples (Figure 4) revealed an extensive biofilm formation with abundant yeast cells, frequently forming aggregates (Figure 2). In exchange a drastic reduction in the biofilm development was observed in the case of textile fibers coated with nanoparticles; these results confirm the quantitative, culture-based assay.

## Conclusions

The optimized textile dressing samples coated with functionalized magnetite nanoparticles proved to be more resistant to *C. albicans* colonization, as compared to the uncoated ones. These functionalized surfaces-based approaches are very useful in the prevention of wounds against microbial contamination and subsequent biofilm development on viable tissues or implanted devices.

## Competing interests

The authors declare that they have no competing interests.

## Authors’ contributions

IA and EA participated in the design and coordination of the study. AMG and MCC conceived the study and drafted the manuscript. AF, VG, and BSV participated in the sequence alignment and performed the synthesis and characterization of the obtained nanoparticles and of the coated materials. CS and AGA performed the microbiological studies. All authors read and approved the final manuscript.
